# BDNF contributes to IBS-like colonic hypersensitivity via activating the enteroglia-nerve unit

**DOI:** 10.1038/srep20320

**Published:** 2016-02-03

**Authors:** Peng Wang, Chao Du, Fei-Xue Chen, Chang-Qing Li, Yan-Bo Yu, Ting Han, Suhail Akhtar, Xiu-Li Zuo, Xiao-Di Tan, Yan-Qing Li

**Affiliations:** 1 Department of Gastroenterology, Qilu Hospital, Shandong University, Jinan 250012, P. R. China; 2 Laboratory of Translational Gastroenterology, Qilu Hospital, Shandong University, Jinan 250012, P. R. China; 3 Department of Physiology, Shandong University School of Medicine, Jinan 250012, P. R. China; 4 Department of Pediatrics, Feinberg School of Medicine, Northwestern University, Chicago, Illinois 60611, USA

## Abstract

The over-expressed colonic brain-derived neurotrophic factor (BDNF) has been reported to be associated with abdominal pain in patients with irritable bowel syndrome (IBS). However, the neuropathological mechanism is unclear. We here investigated the involvement of enteroglial cells (EGCs) and enteric nerves in IBS-like visceral hypersensitivity. We showed that glial fibrillary acidic protein (GFAP), tyrosine receptor kinase B (TrkB) and substance P (SP) were significantly increased in the colonic mucosa of IBS patients. The upregulation of those proteins was also observed in the colon of mice with visceral hypersensitivity, but not in the colon of BDNF^+/−^ mice. Functionally, TrkB or EGC inhibitors, or BDNF knockdown significantly suppressed visceral hypersensitivity in mice. Using the EGC cell line, we found that recombinant human BDNF (r-HuBDNF) could directly activate EGCs via the TrkB-phospholipase Cγ1 pathway, thereby inducing a significant upregulation of SP. Moreover, supernatants from r-HuBDNF-activated EGC culture medium, rather than r-HuBDNF alone, triggered markedly augmented discharges in isolated intestinal mesenteric afferent nerves. r-HuBDNF alone could cause mesenteric afferent mechanical hypersensitivity independently, and this effect was synergistically enhanced by activated EGCs. We conclude that EGC-enteric nerve unit may be involved in IBS-like visceral hypersensitivity, and this process is likely initiated by BDNF-TrkB pathway activation.

Irritable bowel syndrome (IBS) contains a set of challenging symptoms such as abdominal pain or discomfort, bloating and altered bowel habits. IBS afflicts ~15%–24% western and ~5%–10% of Asian populations^1^. Abdominal pain is considered the cardinal feature of IBS, and it seriously affects the quality of life^2^. Visceral hypersensitivity has been recognized as a crucial mechanism underlying pain generation in IBS^3^. Central factors, including enhanced signal perception in the brain and the sensitization of dorsal horn neurons, play a pivotal role in visceral hypersensitivity in IBS^4^. In addition to this classic view, the sensitization of peripheral primary visceral afferent nerves that localize in the gut wall has been demonstrated to be another important origin of abdominal pain in IBS. Emerging human and animal data show significant changes in intestinal mucosa of IBS patients, such as mast-cell activation^5^, increased 5-hydroxytryptamine (5-HT) release^6^ and low-grade inflammation^7^, which might contribute to IBS-like visceral hypersensitivity. However, data in this field are still limited.

The brain-derived neurotrophic factor (BDNF) was originally discovered to play neurotropic and neuroprotective roles in the nervous system^8^,^9^. This cytokine has recently been revealed to be remarkably elevated in dorsal root ganglion (DRG) neurons during neuropathic and inflammatory processes, subsequently leading to pain^10^. Notably, non-neuronal cells such as intestinal epithelial cells also secrete BDNF^11^. We have previously demonstrated that IBS patients have remarkably increased BDNF levels in colonic mucosa. Overexpressed colonic BDNF can be induced by high serine protease activity in the feces of diarrheic IBS patients and is significantly correlated with abdominal pain severity and frequency^12^,^13^. However, the mechanisms of how colonic BDNF modulates the visceral sensation in IBS have not yet been identified.

Enteroglial cells (EGCs) have a morphology and function similar to astrocytes in the brain, and they form an extensive cell network throughout the intestinal wall^14^. The EGC network, connecting the intestinal epithelium and the sub-mucosal enteric nervous system (ENS), has been revealed to play an important role in the maintenance of intestinal homeostasis^15^,^16^. Recent studies have further shown that EGC function can be profoundly changed by many factors, such as pro-inflammatory cytokines^17^, bacteria^18^, and neurotransmitters^19^. Thus, EGCs may undergo dynamic processing under pathological conditions and serve overlapping functions such as modulating intestinal barrier function, mucosal immunity and enteric neurotransmission via releasing various substances^16^. Of interest, tyrosine receptor kinase B (TrkB), the high-affinity receptor of BDNF^20^, has been identified to be expressed in the EGCs of humans^21^ and mice^22^, which implies a possible functional link between BDNF and EGCs. However, whether BDNF-EGC interaction exists and contributes to IBS-like visceral hypersensitivity has yet to be examined. Previous studies have shown that BDNF has no direct effect on the excitability of enteric nerves but can increase their chemical sensitivity^23^,^24^. Whether BDNF can also influence enteric nerve mechanosensitivity remains to be defined.

In this study, we sought to investigate the effect of colonic BDNF on EGC activation and enteric nerve mechanosensitivity to explore the link between BDNF-challenging EGCs, enteric nerves and IBS-like visceral hypersensitivity.

## Results

### Characteristics of study subjects

In the present study, 30 IBS patients (18 women and 12 men; age 49.3 ± 13.4 years) and 30 healthy controls (HCs, 16 women and 14 men; age 44.8 ± 10.4 years) entered this study. In patients with IBS, 66.7% (n = 20) and 33.3% (n = 10) were diagnosed with diarrhea predominant IBS (IBS-D) and constipation predominant IBS (IBS-C), respectively. On the whole, IBS patients had significantly higher BDQ pain severity (*P* < 0.0001) and frequency (*P* < 0.0001) scores than HCs. No difference was found between IBS subgroups in the aspect of the above BDQ scores. The HAD scores between the control group and the IBS group were not significantly different (Table 1). In agreement with previous reports^25^,^26^,^27^,^28^,^29^, fecal serine-protease activities were markedly higher in IBS-D patients (median (IQR) 1521 (905.3–1983) trypsin units/mg protein) than in HCs (465 (264.3–708.0) trypsin units/mg protein; *P* < 0.0001). Aprotinin significantly reduced the protease activities of IBS-D FSN (245 (109.8–413.5) trypsin units/mg protein; *P* < 0.001). In contrast, there were no significant differences in protease activities between IBS-C FSN (455 (253–905.5) trypsin units/mg protein) and HCs.

### In colonic mucosa of IBS patients, EGCs showed higher expression levels of GFAP and TrkB and altered ultrastructure

**Figure 1:**
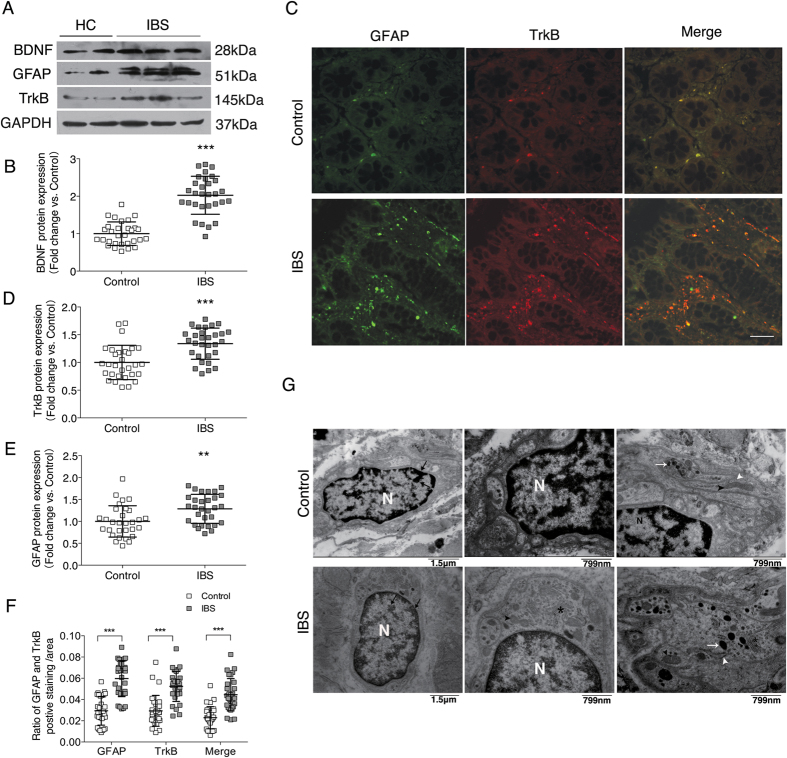
Detection of EGCs in colon of IBS patients and their association with BDNF. (**A,B,D** and **E**) BDNF, GFAP and TrkB protein levels were significantly up regulated in colonic biopsy specimens of IBS patients compared with HCs (The results are displayed as the mean ± SD; ^**^P < 0.01, ^***^P < 0.001. Targeted protein levels were normalized to GAPDH, n = 30 for IBS patients, n = 30 for HCs). (**C**) Co-localization of TrkB and GFAP in colonic mucosa of IBS patients and HCs. (**F**) IBS patients showed increased immunostaining of GFAP and TrkB co-expression in colonic mucosa (***P < 0.001. Scale bars: 50 μm). (**G**) Ultrastructure alterations of mucosal EGCs in colon of IBS patients and HCs. Increased integrated optical density of heterochromatin (black arrow), number of mitochondria (black arrowhead), amount of glycogenosome (white arrow), rough endoplasmic reticulum (black star) and polyribosomes (white arrowhead) appeared in the EGC cell body in the IBS group compared with the HC group (N: nucleus. Scale bars: 799 nm–1.5 μm). The gels were run under the same experimental conditions. Cropped gels/blots are presented. (Full-length gels/blots are shown in Suppl. Fig. S2 with indicated cropping lines)

Consistent with our previous findings^13^, markedly increased BDNF levels in colonic mucosa were confirmed (primarily in epithelial cells and interstitial tissue) in a different population of patients with IBS (Fig. 1A,B; Supplementary Fig. S1A), but there was no difference between IBS-C and IBS-D patients (data not shown). To investigate how BDNF acts in mucosa, we detected TrkB expression. Double immunofluorescent staining identified that TrkB was expressed primarily in EGCs and partially in PGP9.5-immunoreactive nerve fibers (Fig. 1C, Supplementary Fig. S1B). Total TrkB protein levels were significantly upregulated in the colonic mucosal biopsies of patients with IBS (Fig. 1D). Because GFAP is considered as a major marker for astrocyte and EGC activation^30^, we quantified its expression in EGCs. Western blotting and immunostaining confirmed the increased total GFAP (Fig. 1E and Supplementary Fig. S1C) and the co-expression of GFAP and TrkB (Fig. 1F) in colonic mucosal specimens of IBS patients, indicating that TrkB-related EGC activation might exist in IBS patients.

Ultrastructural alterations of EGCs further supported our hypothesis for EGC activation in IBS. Colon-derived EGCs in IBS patients had a decrease in the integrated optical density of heterochromatin (black arrow), which indicated less repression of nearby sequences and more transcriptions^31^, an increased number of mitochondria (black arrow head) and glycogenosome (white arrow), which suggested an elevated protein synthesis (Fig. 1G and Table 2)^32^. In addition, the rough endoplasmic reticulum (black star) and polyribosomes (white arrowhead) also seemed to be increased in the EGC cell body in the IBS group (although they are difficult to quantify).

### GFAP-positive EGCs demonstrated SP-positive immunoreaction and correlated with IBS abdominal pain

**Figure 2:**
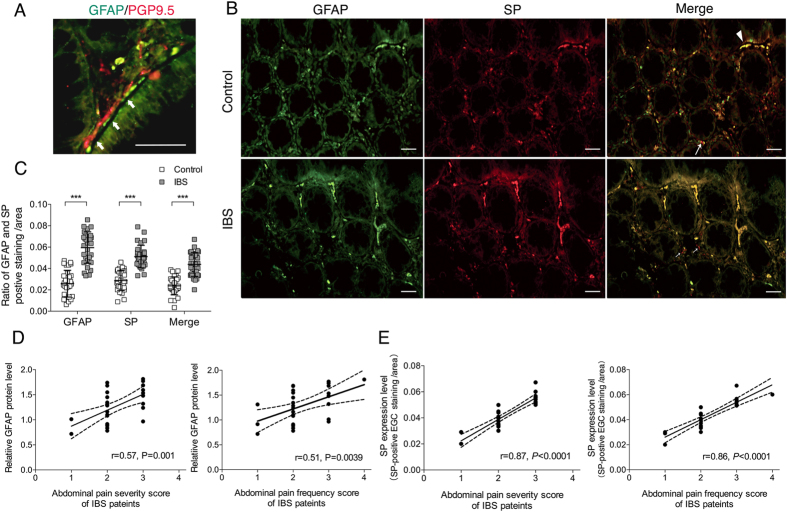
Increased EGCs localize in proximity to colonic nerves, express substance P and correlate with abnormal pain scores in IBS patients. (**A**) Dual immunofluorescence images showing that mucosal EGCs localized in close proximity to mucosal nerve fibers in colon of IBS patients (white arrows; green staining: GFAP; red staining: PGP9.5; scale bars: 50 μm). (**B**) Photomicrographs show GFAP, SP and their co-localization in a HC and IBS rectosigmoid biopsy. White arrowheads refer to the co-expression of GFAP and SP in EGCs. White arrows represent SP-immunoreactive nerve fibers or other cells. (**C**) Semi-quantitative analysis using Image-Pro Plus software revealed that the total SP and EGC-expressed SP immunostainings were both significantly increased in the colonic mucosa of IBS patients compared to HCs. (**D**) Spearman rank correlation between colonic mucosal GFAP protein levels and severity (r = 0.57, *P* = 0.001) and frequency (r = 0.51, *P* = 0.0039) scores of the abdominal pain of patients with IBS. Doted lines refer to 95% CI. (**E**) The Spearman rank correlation between SP-positive EGC immunostaining areas and severity (r = 0.87, *P* < 0.0001) and frequency (r = 0.86, *P* < 0.0001) scores of abdominal pain of patients with IBS. Results are displayed as the mean ± SD. (^***^P < 0.001; n = 30 for IBS patients, n = 30 for HCs; scale bars: 50 μm).

Next we sought to investigate the relationship between EGCs and pain generation. Within ENS such as the sub-mucosal and myenteric plexus, EGCs are revealed to closely accompany nerves^16^,^14^. However, EGCs beneath the gut epithelium are scattered throughout the mucosa, with a complex spatial distribution and potential distinct functions, e.g., promoting mucosal barrier integrity via cross talk with epithelial cells^15^,^16^. Whether EGCs within mucosa exert glia-nerve neurotransmission function is not clear. With dual immunostaining, we found that mucosal EGCs were frequently close to or directly contacted mucosal nerve fibers (Fig. 2A), which provides a structural basis for mucosal glia-nerve interaction.

To determine whether EGCs have the capacity of releasing pain-related substances, we focused on SP. SP is a classic pain signal-transmitting peptide and its increased positive immunoreaction in colonic mucosa of IBS patients has been demonstrated by many studies^33^,^34^. Because SP positive immunoreaction has already been observed in mouse EGCs^35^, we investigated whether it is expressed in human EGCs. As shown in Fig. 2B, a large proportion of SP immunostaining was co-localized with GFAP (white arrow heads) and this co-expression was significantly increased in IBS patients (Fig. 2C). In addition, SP-positive nerve fibers and other cells were also observed in luminal propria (Fig. 2B, white arrows), around EGCs, which was consistent with previous reports^33^,^34^,^36^.

Previously, we found that the increase in BDNF in colonic mucosa is significantly associated with abdominal pain severity and frequency scores in IBS patients. Here, we further examined the correlation between colonic GFAP and SP levels and abdominal pain scores in IBS patients. Relative GFAP protein levels and SP-positive EGC immunostaining areas were categorized according to the corresponding BDQ scores of each IBS patient. As expected, the protein levels of GFAP correlated significantly with abdominal pain severity and frequency scores in IBS patients (Fig. 2D). A similar correlation was observed for SP expression (Fig. 2E). Together, these data provide a foundation for the direct participation of BDNF-EGCs and SP in the process of pain generation in IBS patients.

### IBS-D FSN-mediated visceral hypersensitivity mice over-expressed colonic BDNF, GFAP, TrkB and SP, which were inhibited by BDNF knockdown in BDNF^+/−^ mice.

**Figure 3:**
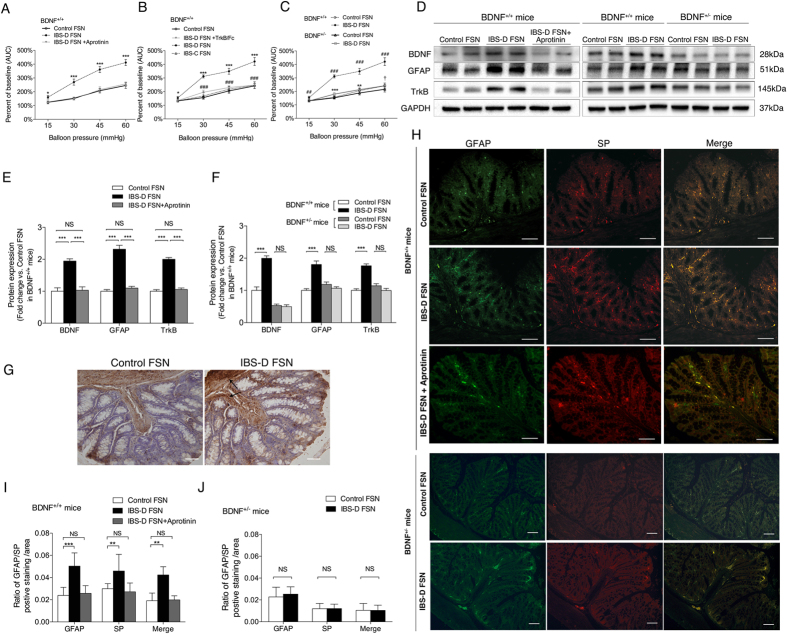
Alterations of visceral sensitivity and expressions of BDNF, GFAP and substance P in colon of BDNF^+/+^ and BDNF^+/−^ mice in the presence or absence of IBS fecal supernatants (FSN) stimulation. (**A**) Intracolonic infusion of IBS-D FSN significantly increased electromyographic (EMG) activity of abdominal muscles in wild-type mice (*, IBS-D FSN vs. control). (**B**) TrkB/Fc significantly suppressed the effect of IBS-D FSN at 30, 45 and 60 mmHg (*, IBS-D FSN vs. control; #, IBS-D FSN vs. IBS-D FSN plus TrkB/Fc). (**C**) The overall response to the CRD after treatment by IBS-D FSN in BDNF^+/−^ mice was significantly lower than that in BDNF^+/+^ mice. (*, IBS-D FSN vs. control FSN in BDNF^+/−^ mice; #, IBS-D FSN in BDNF^+/+^ mice vs. in BDNF^+/−^ mice; ^†^control FSN in BDNF^+/+^ mice vs. in BDNF^+/−^ mice). (**D**–**F**) Western blotting analysis of BDNF, GFAP and TrkB protein levels in colon of BDNF^+/+^ and BDNF^+/−^ mice 4 h after intracolonic infusion with IBS-D FSN compared with control FSN or aprotinin-pretreated IBS-D FSN. (**G**) Overview of GFAP expression in the full-thickness colonic wall in BDNF^+/+^ mice 4 h after intracolonic infusion with IBS-D FSN compared with control FSN. Black arrow shows increased expression of GFAP in muscular layers. (**H**) Dual immunofluorescence staining of SP and GFAP in colon of BDNF^+/+^ and BDNF^+/−^ mice 4 h after intracolonic infusion with IBS-D FSN compared with control FSN or aprotinin-pretreated IBS-D FSN. Co-immunostaining for colonic SP and GFAP was significantly increased in BDNF^+/+^ mice (**I**) but not in BDNF^+/−^ mice (**J**) after 4-hour intracolonic incubation with IBS-D FSN. The results are displayed as the mean ± SD. (^*^P < 0.05 ^**^P < 0.01, ^***^P < 0.001, ^##^*P* < 0.01, ^###^*P* < 0.001, ^†^*P* < 0.05; AUC, area under curve; NS, not significant; target protein levels were normalized to GAPDH; n = 8 per group; scale bars: 50 μm). The gels were run under the same experimental conditions. Cropped gels/blots are presented. (Full-length gels/blots are shown in Suppl. Fig. S2 with indicated cropping lines.)

It has been reported that IBS-D FSN has an increased serine-protease activity and can rapidly lead to increased paracellular permeability and hypersensitivity in mice colon, likely through activating protease-activated receptor-2 (PAR-2)^25^,^26^. This stimulus to a large extent mimics the luminal environment of IBS patients. Thus, we selected this visceral hypersensitivity animal model to reproduce the changes in colonic BDNF and GFAP expression during the hypersensitivity-inducing process. In our study, we also showed a remarkably increased serine-protease activity in IBS-D FSN. Participants with serine-protease activity higher than the maximum value (1436 U/mg protein) in the HC and IBS-C groups were selected for inducing mouse visceral hypersensitivity. As shown in Fig. 3A,B, the EMG responses were significantly increased under colorectal distension (CRD) pressures of 15, 30, 45, and 60 mmHg at 4 h after infusion with IBS-D FSN in BDNF^+/+^ mice, compared with mice treated with HC FSN or aprotinin-pretreated IBS-D FSN. This finding suggests that FSN from our cohort of IBS-D patients also experienced a sensitizing effect, and this effect should be produced by the increased serine protease activity in IBS-D FSN. Furthermore, the increased response to CRD was prevented under 30, 45 and 60 mmHg pressures when mice were pretreated with TrkB/Fc, indicating that a TrkB-mediated signaling pathway participated in IBS-D FSN-induced visceral hypersensitivity. No other abnormal behavioral effects of TrkB/Fc or aprotinin were detected. IBS-C FSN did not cause hypersensitivity after single infusion, which was consistent with the reports of Gecse, K and Annahazi^25^,^27^.

We utilized BDNF^+/−^ mice to further verify whether BDNF is involved in the colonic hypersensitivity-inducing process. As illustrated in Fig. 3C, the EMG responses under CRD pressures of 15, 30, 45, and 60 mmHg were markedly lower in BDNF^+/−^ mice than in BDNF^+/+^ mice after IBS-D FSN infusion, and IBS-D FSN even failed to induce visceral hypersensitivity in BDNF^+/−^ mice under 15 and 60 mmHg CRD pressures when compared with HC FSN. In addition, the EMG responses did not change much under 15 and 30 mmHg between BDNF^+/+^ and BDNF^+/−^ mice after HC FSN infusion. Lower VMR scores were observed in BDNF^+/−^ mice at 45 mmHg (*P* = 0.083, not statistically significant) and 60 mmHg pressures (*P* <0.05), which indicated that BDNF knockdown did not influence much on basal visceral sensitivity with a CRD pressure <60 mmHg under controlled FSN stimulation. Collectively, these data suggest that the mechanism of IBS-D FSN-induced hypersensitivity may involve a BDNF-TrkB signaling pathway.

To investigate the association between BDNF, EGC activity and visceral hypersensitivity, we examined alterations of colonic BDNF, TrkB and GFAP protein levels in mice with IBS-D FSN-induced visceral hypersensitivity and mice with BDNF^+/−^. As shown in Fig. 3D–F, the BDNF levels were significantly increased in the colon of BDNF^+/+^ mice after intracolonic incubation with IBS-D FSN compared with HC FSN or aprotinin-pretreated IBS-D FSN. In BDNF^+/−^ mice, colonic BDNF levels dropped by nearly half, and did not change under IBS-D FSN stimulation compared with HC FSN. In parallel with alterations in BDNF levels, GFAP and TrkB expressions in colon were also significantly increased in IBS-D FSN-treated BDNF^+/+^ mice. Immunohistochemistry staining further showed that the increased GFAP expression in IBS-D FSN-treated wide type mice appeared not only in mucosa but also throughout the full-thickness colonic wall (Fig. 3G). However, in BDNF^+/−^ mice, GFAP and TrkB levels remained almost unchanged when treated with either IBS-D or control FSN (Fig. 3D–F). Similarly, total SP and EGC-expressed SP immunoreaction in the colon of BDNF^+/+^ mice were also both expectedly increased after the intracolonic infusion of IBS-D FSN, but they were unchanged (even decreased compared with BDNF^+/+^ mice) in BDNF^+/−^ mice (Fig. 3H–J). Taken together, these data suggested that the mechanism of IBS-D FSN-induced visceral hypersensitivity in mice is BDNF dependent. Within the colon, BDNF seems to be crucial for EGC activation and SP production.

### EGC dysfunction prevented effect of IBS-D FSN-induced visceral hypersensitivity and SP production in mice

**Figure 4:**
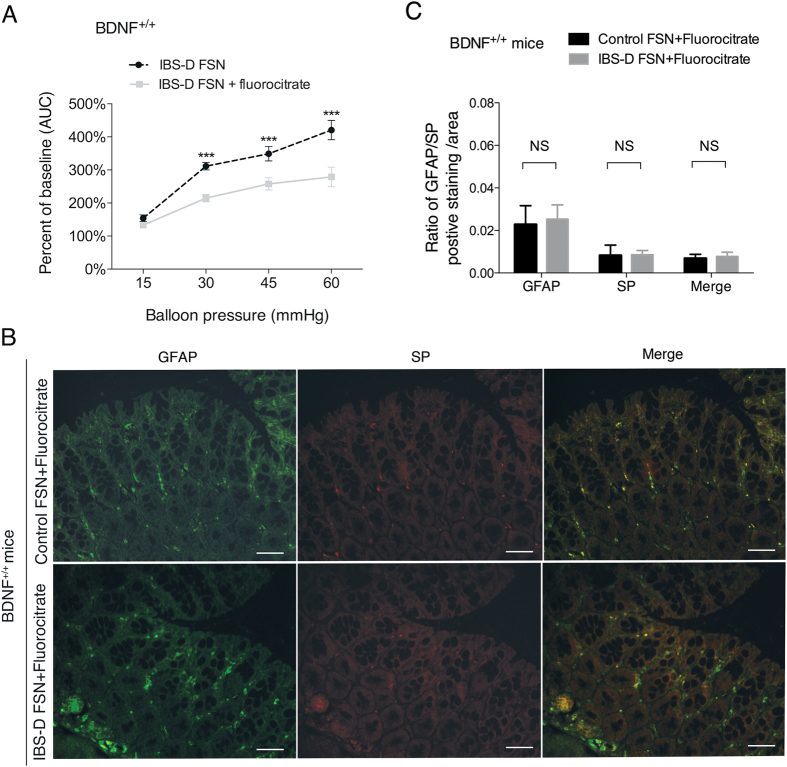
Effect of EGC dysfunction on visceral sensitivity and SP production. **(A)** Fluorocitrate-mediated dysfunction of EGCs significantly suppressed the mouse abdominal contraction response triggered by IBS-D FSN at CRD pressures of 30, 45 and 60 mmHg (^***^P < 0.001). (**B**,**C**) dual immunofluorescence staining showed that IBS-D FSN failed to upregulate the SP production of EGCs in fluorocitrate-treated mice (NS, not significant). Data are displayed as the mean ± SD. Scale bars: 50 μm.

Next we specifically destructed the normal function of EGCs in mice using fluorocitrate to confirm the role of EGCs in visceral hypersensitivity. Previous administration of fluorocitrate in mice significantly decreased the elevated visceromotor response (VMR) scores evoked by IBS-D FSN under CRD pressures of 30, 45 and 60 mmHg (Fig. 4A). In addition, IBS-D FSN failed to evoke an increase in SP immunostaining in EGCs (Fig. 4B,C).

### r-HuBDNF directly activated rat EGC cell line (CRL-2690 cells) to produce SP via TrkB-dependent activation of PLCγ1 signaling pathway

**Figure 5:**
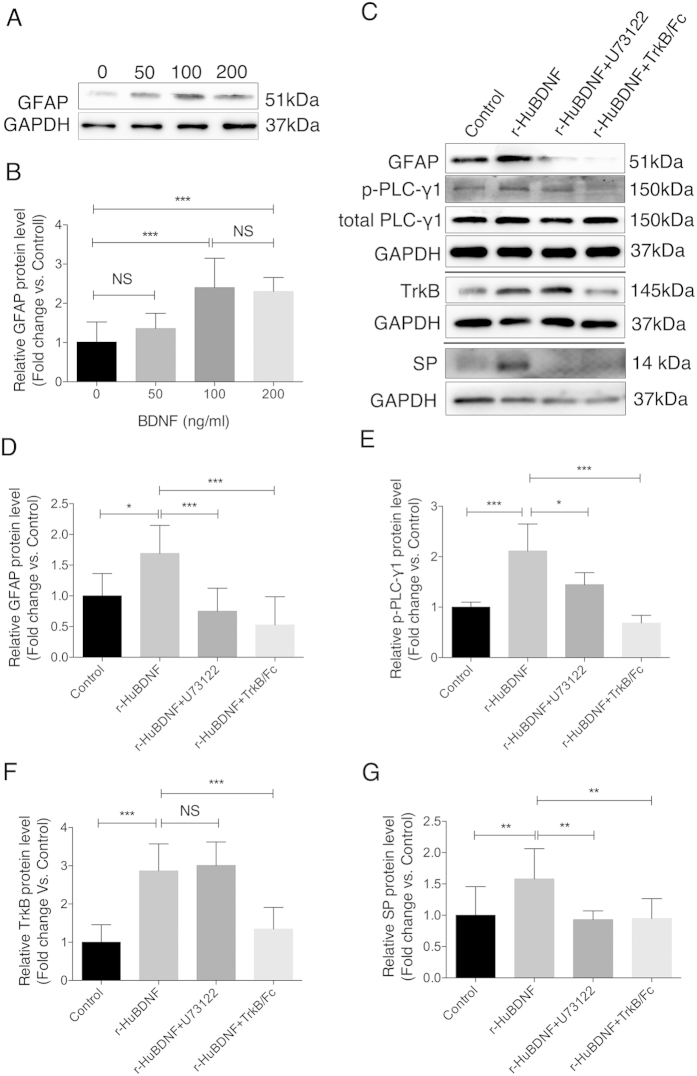
r-HuBDNF directly activated rat EGC cell line (CRL-2690) via a TrkB-PLCγ1 phosphorylation pathway. (**A**,**B**) r-HuBDNF elevated expression of GFAP in CRL-2690 cells dose-dependently. GFAP levels reached the maximum at 100 ng/ml of r-HuBDNF challenging for 2 h. (**C**–**G**) Western blotting detected expression of GFAP, TrkB, p-PLCγ1, total PLCγ1 and SP proteins in CRL-2690 cells treated by r-HuBDNF (100 ng/ml, 2 h) alone or U73122 or TrkB/Fc-pretreatment followed by r-HuBDNF (100 ng/ml, 2 h) challenging. r-HuBDNF-induced up-regulations of GFAP, TrkB, p-PLCγ1 and SP were significantly attenuated by U73122 or TrkB/Fc. Data are displayed as the mean ± SD of 3 separate experiments. (^*^P < 0.05, ^**^P < 0.01, ^***^P < 0.001; NS, not significant. Target protein levels were normalized to GAPDH; no difference for total PLCγ1 between groups. For the SP detection experiment, cells were co-incubated with Brefeldin A, 3 ug/ml, 2 h.) The gels were run under the same experimental conditions. Cropped gels/blots are presented. (Full-length gels/blots are shown in Suppl. Fig. S2 with indicated cropping lines.)

Next we examined whether BDNF could directly activate EGCs *in vitro*. To do so, CRL-2690 cells (a rat EGC cell line) were treated with r-HuBDNF for 2 h. We found that r-HuBDNF significantly increased the expression of GFAP in a dose-dependent manner (Fig. 5A,B). To determine how r-HuBDNF triggered GFAP expression in EGC cells, we further studied the PLCγ1 (a major substrate of TrkB) signaling pathway, which plays a key role in modulating intracellular Ca^2+^ activity^37^. For this purpose, EGC cells were pretreated with TrkB/Fc (an antagonist of TrkB) or U73122 (a PLCγ1 inhibitor) for 30 min. Then, the cells were stimulated with r-HuBDNF (100 ng/ml), followed by measuring the expression of GFAP, TrkB, PLCγ1 and tyrosine (Tyr-783)-phosphorylated PLCγ1 with immunoblotting. As demonstrated in Fig. 5C–F, TrkB/Fc completely blocked the r-HuBDNF-induced expression of GFAP and TrkB and the phosphorylation of PLCγ1. U73122 was also found to markedly inhibit the r-HuBDNF-induced phosphorylation of PLCγ1 and GFAP expression.

To confirm whether SP is expressed in BDNF-activated rat EGCs, we next detected SP protein levels in CRL-2690 cells. As shown in Fig. 5G, untreated CRL-2690 cells expressed a very low level of SP. In contrast, r-HuBDNF significantly upregulated SP protein levels compared with control in CRL-2690 cells, and U73122 and TrkB/Fc could completely inhibit the expression of SP. Thus, these data suggested that BDNF could directly activate rat EGCs to secrete SP, which might be regulated by the TrkB-PLCγ1 pathway.

### Supernatants from r-HuBDNF-treated rat EGC culture medium but not r-HuBDNF itself, enhanced discharge of mesenteric afferent nerves

**Figure 6:**
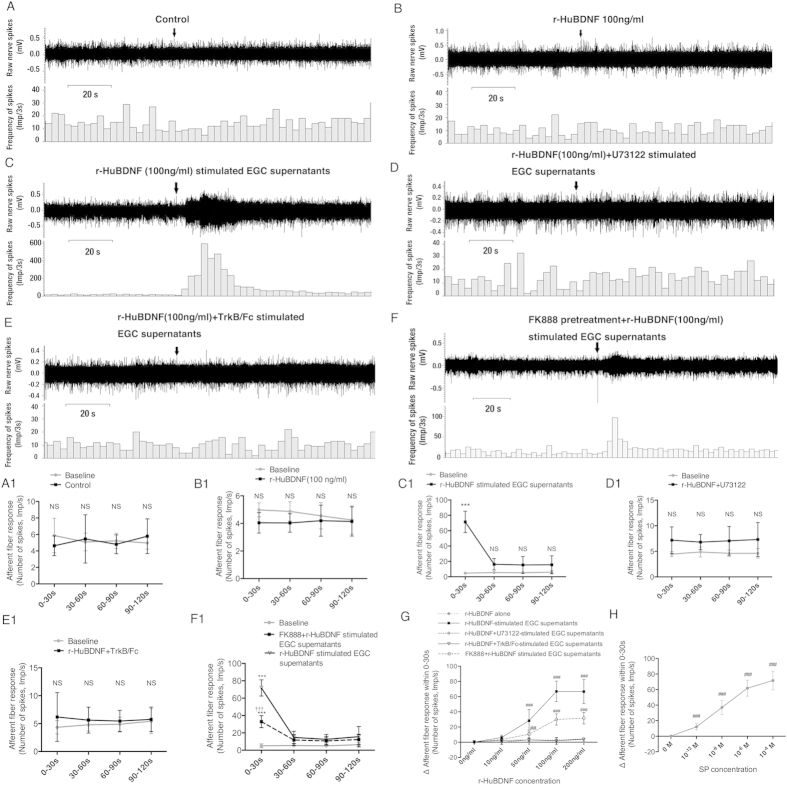
Culture supernatants obtained from r-HuBDNF-challenged CRL-2690 cells triggered an increased discharge of rat mesenteric afferent nerves. (**A** and **A1**) Control culture supernatants from untreated CRL-2690 cells had no excitatory effect on rat mesenteric afferent nerves. (**B** and **B1**) r-HuBDNF alone did not induce any enhanced discharge of mesenteric afferent nerves either. (**C** and **C1**) supernatants from r-HuBDNF-challenged CRL-2690 cells significantly evoked the discharge of rat mesenteric afferent nerves during the first 30 seconds of treatment on the basis of the baseline. Supernatants obtained from r-HuBDNF-challenged CRL-2690 cells in the presence of U73122 (**D** and **D1**) or TrkB/Fc (**E** and **E1**) did not increase any discharge of mesenteric afferent nerves. (**F** and **F1**) Preincubation of mesenteric afferent nerves with FK888 (4 × 10^−6 ^M, 15 min) substantially reduced the ability of r-HuBDNF-stimulated EGC supernatants to induce fiber discharge; however, a residual excitatory effect remained (0–30s). (**G**) Dose-response curve showed effects of r-HuBDNF on discharge of mesenteric afferent nerves at different concentrations. r-HuBDNF had a dose-dependent effect on the ability of EGC supernatants to increase nerve discharge. r-HuBDNF alone had no excitatory effect at any dose. TrkB/Fc and U73122 completely, whereas FK888 partially blocked the excitatory effect of r-HuBDNF-stimulated EGC supernatants. (**H**) Dose-response curve showed that SP could trigger an increased discharge of mesenteric afferent nerves at either a low or a high concentration. Data are displayed as the mean ± SD. (^***^P < 0.001, NS, not significant compared with baseline; ^##^*P* < 0.01, ^###^*P* < 0.001, compared with control supernatants; ^†††^*P* < 0.001, FK888 + r-HuBDNF-stimulated EGC supernatants vs. r-HuBDNF-stimulated EGC supernatants. n = 5 per group.)

Next, we performed a mesenteric afferent nerve recording assay to ascertain whether BDNF-activated EGCs have a practical excitatory effect on nerve fibers. We found that control culture supernatants had no effect on the nerve discharge rate in any duration of time (Fig. 6A,A1). Because it is difficult to determine what concentration of r-HuBDNF would be “physiologically” appropriate, we created a concentration-response curve to validate the direct effect of r-HuBDNF on afferent discharge. The concentration-response curve showed that r-HuBDNF alone (10–200 ng/ml) had no excitatory role in afferent discharge compared with the baseline (Fig. 6B,B1). In sharp contrast, supernatants collected from r-HuBDNF (50~200 ng/ml)-treated CRL-2690 cells significantly increased the afferent discharge during the first 30 seconds (Fig. 6C,C1). However, when intervened with U73122 or TrkB/Fc, supernatants from r-HuBDNF-treated EGCs could not induce any augmentation of afferent discharge on the basis of the baseline (Fig. 6D,D1). To further confirm the role of SP in triggering nerve firing, we preincubated the mesenteric nerves with FK888 (4 × 10^−6^ M, 15 min) to block the SP receptor NK1. We found that FK888 significantly inhibited the excitatory effect of r-HuBDNF-treated EGC supernatants. Notably, a residual effect of r-HuBDNF-treated EGC supernatants remained and was significantly greater than the baseline measurements, which suggested that other untested mediators secreted by activated EGCs also had potential effects on mesenteric nerve firing (Fig. 6F,F1). In addition, the concentration-response curve showed that both low and high levels of SP (10^−12^–10^−4^ M) could significantly enhance the afferent discharge (Fig. 6H), indicating SP is a strong inducer of nerve firing. Collectively, we propose that BDNF-stimulated rat EGCs have a significant excitatory effect on sensory nerves, likely through releasing multiple amounts of excitatory neurotransmitters including SP.

### r-HuBDNF increased mechanosensitivity of mesenteric afferent nerves, which was significantly enhanced by EGC activation

**Figure 7:**
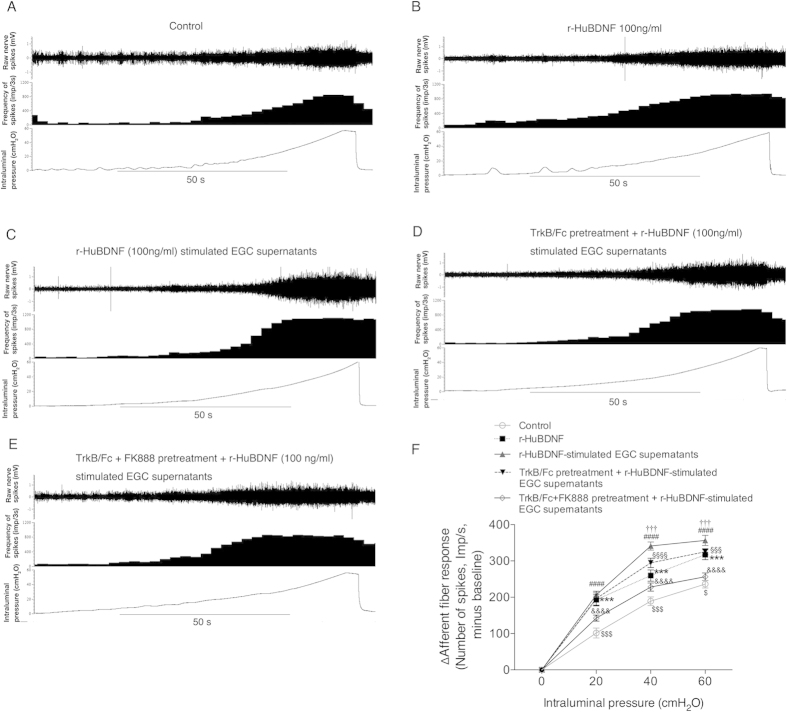
r-HuBDNF and r-HuBDNF-activated EGCs exert synergistic effects on increasing rat mesenteric afferent mechanosensitivity. Representative recordings of jejunal mesenteric afferent discharge to luminal ramp distension after preincubation with different stimuli (15 min): (**A**) control supernatants; (**B**) r-HuBDNF alone (100 ng/ml); (**C**) r-HuBDNF (100 ng/ml)-stimulated EGC supernatants; (**D**) TrkB/Fc (2.5 μg/ml) pretreatment followed by administration with r-HuBDNF-stimulated EGC supernatants; (**E**) TrkB/Fc (2.5 μg/ml) + FK888 (4 × 10^−6^ M) pretreatment followed by administration with r-HuBDNF-stimulated EGC supernatants. (**F**) Summary for mesenteric afferent discharge to luminal ramp distension minus baseline. ^***^P < 0.001, r-HuBDNF alone vs. control; ^####^*P* < 0.0001, r-HuBDNF-stimulated EGC supernatants vs. control; ^†††^*P* < 0.001, r-HuBDNF-stimulated EGC supernatants vs. r-HuBDNF alone; ^§§§^*P* < 0.001, ^§§§§^*P* < 0.0001 TrkB/Fc pretreatment + r-HuBDNF-stimulated EGC supernatants vs. r-HuBDNF-stimulated EGC supernatants; ^&&&&^*P* < 0.0001, TrkB/Fc + FK888 pretreatment + r-HuBDNF-stimulated EGC supernatants vs. r-HuBDNF-stimulated EGC supernatants; ^$^*P* < 0.05, ^$$$^*P* < 0.001, TrkB/Fc + FK888 pretreatment + r-HuBDNF-stimulated EGC supernatants vs. control. Data are displayed as the mean ± SD; n = 5 per group.

Finally, we examined whether r-HuBDNF alone or r-HuBDNF-stimulated EGC supernatants would affect the action potential firing in response to mechanical stimulation. With mesenteric afferent nerve recording assay, we found that in comparison to control supernatants (Fig. 7A), supernatants from r-HuBDNF (100 ng/ml)-treated EGCs induced the greatest increase in the mechanosensitivity of mesenteric afferent nerves (Fig. 7C,F). Modest, but significant increases in mechanosensitivity were also observed following pretreatment with r-HuBDNF (100 ng/ml) alone (Fig. 7B,F). By contrast, preincubation of the nerve with TrkB/Fc (2.5 ug/ml) partially repressed the increase induced by r-HuBDNF-stimulated EGC supernatants (Fig. 7D,F), whereas preincubation with TrkB/Fc and FK888 together remarkably inhibited the increase in mechanosensitivity caused by rHuBDNF-stimulated EGC supernatants (Fig. 7E,F). Collectively, these data suggest that r-HuBDNF alone can cause the mechanosensitivity of intestinal afferent nerves but although not directly induce nerve discharge. r-HuBDNF-activated EGCs also can increase nerve mechanosensitivity through secreting multiple mediators including SP. Thus, r-HuBDNF and activated EGCs may exert a synergistic effect in enhancing the mechanical sensitivity of afferent nerves.

## Discussion

Our study demonstrates that increased colonic BDNF may contribute to visceral hypersensitivity in IBS patients, likely via interacting with the EGC-enteric nerve unit. First, we found that GFAP, an EGC activation marker, together with TrkB and SP expressed by EGCs significantly increased in the colonic mucosa of IBS patients and the increased GFAP and SP are positively correlated with abdominal pain scores. Second, using mice with IBS-D FSN-induced visceral hypersensitivity and mice with BDNF^+/−^, together with mesenteric afferent nerve recording, we found that EGC activation, which is BDNF dependent, is involved in the inducing process of visceral hypersensitivity, and BDNF alone can also act synergistically with EGC through increasing the sensitivity of enteric nerve to chemical or mechanical stimulation. Moreover, using an EGC cell line, we report that TrkB-PLCγ1 pathway may mediate BDNF-induced EGC activation.

BDNF has been recognized as an important pain modulator in the central nervous system (CNS)^38^. In the gut, the actions of BDNF have been revealed to enhance the peristaltic reflex and promote motility^23^,^39^. Other functions, however, are still largely unknown. Our previous data showing association between colonic BDNF and IBS abdominal pain indicate a basis for BDNF in modulating colonic hypersensitivity^13^. Because BDNF primarily acts through its high-affinity receptor TrkB, to illuminate the function mode of colonic BDNF in IBS-like hypersensitivity, we first confirmed that TrkB was primarily expressed in EGCs and the enteric nerve fibers of human colon. This finding is in agreement with previous reports, although other mucosal cell types cannot be ruled out^21^,^22^. Therefore, there are at least three possible mechanisms for colonic mucosal BDNF affecting visceral hypersensitivity: (i) direct effect on enteric nerve fibers, (ii) action through interacting with EGCs, and (iii) action via other mucosal cells. Herwe here primarily focused on hypothesis (i) and (ii). We showed that r-HuBDNF alone did not induce any discharge of isolated mesentery afferent nerves at either low or high concentrations but significantly increased nerve mechanosensitivity. Although this experiment was performed on rat nerves, the results are in line with previous reports that BDNF does not directly activate the signaling of pig ENS but enhances the excitatory effects induced by other neurotransmitters^23^,^24^, suggesting a functional consistency of BDNF in intestines among different species. Therefore, it seems that intestinal BDNF alone is unlikely to induce spontaneous pain in IBS, but it likely contributes to increased nerve chemical and mechanical sensitivity.

To further examine whether BDNF also modulates IBS abdominal pain through other pathways, we investigated the role of BDNF in EGC activity. In CNS, BDNF has been demonstrated to directly activate astrocytes and microglia, which in turn promote the release of proinflammatory factors^40^,^41^. Similarly, our current results for the increased GFAP and TrkB expression in the EGCs of IBS patients, along with the TEM data, provide an important clue for the enhanced BDNF-EGC interaction. To confirm this, we established a visceral hypersensitivity mouse model by performing the intracolonical infusion of IBS-D FSN. We did not choose other models such as the TNBS colitis model because it is known to have an obvious inflammatory process, which may activate EGCs via immune activation^17^, or the stress-induced hypersensitivity model because the upregulation of BDNF in CNS might intervene in our study on the colon^42^. We also did not choose IBS-C FSN as a stimulus because of its delayed sensitizing effect. (Repeated intracolonic infusion is necessary.)^27^. In the current model, we showed that IBS-D FSN could significantly upregulate BDNF, TrkB, GFAP and SP levels in colon, whereas knockdown of colonic BDNF in BDNF^+/−^ mice markedly prevented these increases, suggesting that BDNF likely contributes to EGC activation in this hypersensitivity mouse model.

The direct effect of BDNF on EGCs was examined *in vitro*. In a rat EGC cell-line system, administration of r-HuBDNF significantly induced the upregulation of TrkB, GFAP and SP protein levels. To obtain more mechanistic insights into BDNF and EGC responses, we investigated PLCγ1, which is one of TrkB substrates and directly binds to TrkB via the internal SH2-domain^37^,^43^. Furthermore, PLCγ1 has been found to be expressed in astrocytes and activated through a phosphorylation process^44^. In agreement with these reports, we revealed a similar role of PLCγ1 in EGC activation: r-HuBDNF significantly increased the phosphorylation levels of PLCγ1 in rat EGC cells, which were partially suppressed by U73122 and completely blocked by TrkB inhibitor. This finding suggests that TrkB may serve as a “valve” to control the BDNF action in EGC activation. Additionally, inhibitors of either TrkB or PLCγ1 showed its effect on blocking the r-HuBDNF-induced upregulation of GFAP expression and repressing the release of SP, which further confirmed the important role of TrkB-PLCγ1 in regulating EGC activation.

Whether activated EGCs have a practical excitatory and sensitizing role in sensory nerves is a key point of our hypothesis. In human colonic mucosa, we found that EGCs closely localized in proximity to mucosal nerve fibers, such as mast cells^5^, which may facilitate their functional link to nerves on a morphological basis. Because SP has been revealed to induce the spontaneous discharge of normal or injured nerves^45^,^46^, and we also confirmed that SP could remarkably trigger the augmentation of the discharge of rat intestinal mesenteric afferent nerves even at a very low level, these data suggest that SP plays an important role in enhancing colonic sensory nerve excitability. Along this line, we provide substantial evidence of the capacity of EGCs to produce SP, which is BDNF dependent. First, we confirmed SP expression in human EGCs. Both total SP and EGC-specific SP immunoreactive stainings were significantly increased in the colonic mucosa of IBS patients, in line with previous reports^33^,^34^. Second, we also observed an increased immunoreaction of SP-positive colonic EGCs in mice with IBS-D FSN-induced visceral hypersensitivity but not in BDNF^+/−^ mice, further suggesting that SP-producing EGCs may be involved in the mechanisms of visceral hypersensitivity, likely dependent on the BDNF-associated pathway. Third, direct evidence was obtained from the rat EGC-cell line study. We found that r-HuBDNF-stimulated CRL-2690 cells expressed more SP than unchallenged cells. Together, these data are in agreement with previous reports that mouse EGCs and astrocytes can secrete SP^35^,^47^, and this SP-producing process is shown to be correlated to the BDNF signaling pathway. Ultimately, mesenteric afferent nerve recording verified the practical effect of BDNF-activated EGCs on sensory nerve excitability and mechanosensitivity via SP. First, the discharge of nerves was remarkably reinforced after adding the r-HuBDNF-stimulated EGC supernatants, which was significantly inhibited by the NK1 antagonist, suggesting that r-HuBDNF-activated EGCs can induce spontaneous discharge on sensory nerves. Second, the increased action potential firing of nerves in response to mechanical stimulation after incubation with r-HuBDNF-activated EGC supernatants, which can be blocked by TrkB and NK1 antagonists, indicates that activated EGCs also contribute to nerves’ mechanical hypersensitivity.

Because noxious signals from the colon are generally transmitted via splanchnic neurons, and the nociceptors in the gut wall are primarily located in the muscularis externa and serosa^48^, the question of how noxious signals transmit from mucosa to the serosa needs to be addressed. In IBS-D FSN-stimulated mice colon, we observed not only that sub-epithelial EGCs were activated, but that EGCs within submucous and muscularis externa were also involved (showing significantly increased GFAP immunostaining). This finding suggests that activation signals on EGCs might transmit from mucosa to ENS. This event is expected to amplify the activation signals through the activation of the TrkB-PLCγ1 pathway followed by an increase in the intercellular calcium waves from one EGC to surrounding EGCs in the intestinal wall^49^. Thus, the activated EGCs within deeper layers may help transmitting noxious signals initiated from mucosa to muscularis externa and serosa to activate nociceptors, thereby sensitizing splanchnic nerves.

Fluorocitrate is a metabolic inhibitor that specifically causes the functional disruption of astrocytes but not neurons at certain concentrations^50^. According to Yasmin’s experience^51^, intraperitoneal injections of fluorocitrate can specifically cause mouse EGC dysfunction without inducing intestinal inflammation or affecting spinal astrocytes. Here, we found that 20 μmol/kg fluorocitrate significantly inhibited the effect of IBS-D FSN on evoking visceral hypersensitivity. Moreover, SP-positive immunostaining localized in EGCs was markedly reduced although GFAP expression was not obviously affected, indicating that the production of SP in EGCs was repressed during fluorocitrate-induced EGC dysfunction. These results further support our hypothesis of an EGC activation-mediated pathway in visceral hypersensitivity. Using BDNF^+/−^ and TrkB/Fc-pretreated mice, we provide evidence that overall BDNF levels are responsible for visceral hypersensitivity but still cannot clearly distinguish the role of BDNF specifically expressed in the spinal cord or colon in visceral hypersensitivity. To achieve this goal, further research using intestinal epithelium-specific BDNF knockout mice needs to be conducted. However, we here established a causal relationship between colonic BDNF and EGC activation and an association between EGC activation and visceral hypersensitivity, which could support the concept of the BDNF-EGC-sensory nerve sensitization pathway in colon.

Our study has some limitations. First, our main hypothesis was based on an observational study in humans and then tested in animal and cell experiments. The major limitation is that these preclinical results cannot exactly or completely explain the real pathophysiological changes in humans with IBS. Second, the sample size for the human study is relatively small, which may undermine the accuracy of our results. Thus, further research with a larger number of IBS patients and controls should be conducted to verify our conclusions.

In summary, we demonstrated that GFAP, an EGC activation marker, was significantly increased in the colonic mucosa of IBS patents. Studies on mouse and rat EGCs suggest that EGC activation may be involved in IBS-like visceral hypersensitivity independently or synergistically with BDNF, and this process may be triggered via a BDNF-TrkB-associated signaling pathway. Thus, our study specifically addressed the potential mechanism of visceral hypersensitivity mediated by colonic BDNF in IBS^52^. This study lays a basis to further considering BDNF and the EGC-enteric nerve unit as a potential therapeutic target for IBS abdominal pain.

## Methods

### Patients

All subjects gave their written informed consent prior to enrollment, and the clinical ethical committee of the Qilu Hospital of Shandong University approved the study protocol (Document No. 12113). All methods used in the human study were carried out in accordance with the approved guidelines. IBS patients and age- and sex-matched control subjects were invited to participate in this study at the Department of Gastroenterology at Qilu Hospital. The diagnosis of IBS, based on the Rome III criteria, was further sub-classified as having IBS-D or IBS-C IBS. HCs were defined as individuals who were undergoing colorectal cancer screening and had negative results. The sample size was determined in reference to the results of our previous studies and similar observational studies on IBS^12^,^13^,^53^. We excluded patients with post-infectious IBS because this group of patients indeed has an inflammatory process, which might lead to a bias. Patients with mixed IBS (IBS-M) were also not evaluated because of the very limited enrollment number in the present study phase. Furthermore, none of the enrolled patients or HCs was taking non-steroidal anti-inflammatory drugs, histamine antagonists, mast cell stabilizers, antibiotics, probiotics, tricyclic antidepressant or serotonin selective reuptake inhibitors, steroids, or any pain killers, had undergone major abdominal surgery or had any organic gastrointestinal disorders, including inflammatory bowel disease, coeliac disease, allergic diseases or psychiatric disorders. (Each subject completed a Hospital Anxiety and Depression Scale; HAD scale^54^). Other symptoms specific to females, such as chronic pelvic pain syndrome, dysmenorrhea and endometriosis, were excluded. Each IBS patient was invited to complete an Italian modified version of the Bowel Disease Questionnaire (BDQ) to evaluate gastrointestinal symptoms^55^. (Supplementary Methods-1).

### Fecal supernatants (FSN) and biopsies collection

Fecal samples were collected from HCs and IBS patients before bowel preparation for colonoscopy, *in situ* or at home, and then transported to the Department of Gastroenterology of Qilu Hospital within 1 hour. Samples were dissolved in 0.9% NaCl solution (1 g sample/7 ml saline for mice) and homogenized on ice. After centrifugation (4500 rpm, 4 °C, 10 min), supernatants were collected, filtered on 0.8 μm-sized filters, and then stored at −20 °C. Before mouse experiments, serine-protease activities of all samples were tested according to Gecse’s protocol^25^ (Supplementary Methods-2). To validate most fecal protease is serine-protease, IBS-D FSN was preincubated with serine protease inhibitor aprotinin (100 μg/ml) for 30 min, prior to following protease activity measurements. Protease activity was normalized against protein content and expressed as trypsin activity units/mg protein.

All participants underwent a colonoscopy after a standard bowel preparation using polyethylene glycol. We took 6 specimens for each participant from the rectosigmoid junction (standardizing the site of sampling). Two biopsies were used for routine hematoxylin and eosin histology, immunohistochemistry and immunofluorescence; two biopsies were used for western blotting; and two specimens were used for transmission electron microscopy (TEM).

### Immunohistochemistry and western blotting for human specimens

Immunohistochemistry was used for detecting the distribution and overall expression of BDNF and glial fibrillary acidic protein (GFAP). Double immunostaining was used for detecting TrkB and substance P (SP) expressed in EGCs^56^ or on nerve fibers. Western blotting was used for quantifying the expression of BDNF, GFAP and TrkB proteins in biopsy specimens (Supplementary Methods-3).

### TEM

TEM was used to study the morphological changes in EGCs. (See detailed methods in Supplementary Methods-4). The quantification of the ultrastructure changes in EGCs are as follows: The integrated optical density of heterochromatin that reflects the transcription status of the cells was calculated using Image-Pro 6.0 Plus software. The numbers of mitochondrion and glycogenosome were counted to evaluate the protein synthesis of the cells, and the mean value was obtained for each individual. To minimize subjective bias, two experienced investigators (FXC and YBY), who were both blinded to the clinical and endoscopic information of the patients, performed the morphometric analysis (Evaluation was repeated after two weeks).

### Animals

The protocol of the animal experiments were approved by the Animal Care and Use Committee of Shandong University (Document No. 201202023). All experimental procedures were carried out in accordance with the Animal Management Rules of the Chinese Ministry of Health (Document No. 55, 2001) and the guidelines of the International Association for the Study of Pain.

Male heterozygous BDNF^+/−^ mice (C57BL/6 background) and BDNF^+/+^ littermates (gifts from the Neurobiology Laboratory of Shandong University, genetic status confirmed by PCR) at 4 months of age were used for visceral sensitivity studies. Male wild-type Wistar rats (gifts from the Key Lab of Mental Disorder of Shandong Province, Shandong University), 280–300 g, were used for mesenteric afferent nerve recording studies. All animals were maintained in transparent plastic cages in a temperature-, light- and hygrometry-controlled room (20 ± 2 °C, 12: 12 h light/dark cycle, 50 ± 5%) at the key Laboratory of Cardiovascular Remodeling and Function Research, Qilu Hospital of Shandong University. During the experiment, animals were allocated at random by one investigator.

#### Intracolonic infusion, colorectal distension (CRD) and electromyography recording (EMG)

Silver electrode implanted mice were prepared before all mice experiments according to Christianson’s methods^57^ (Supplementary Methods-5). All treated mice had 5 days for recovery and fasted for 24 h before CRD and EMG detection.

BDNF^+/+^ and BDNF^+/−^ mice received an intracolonic infusion of FSN (0.3 ml, 170 μl/h) from HCs and IBS-D (with or without aprotinin preincubation) or IBS-C patients through a catheter (outside diameter 1 mm) inserted into the colon 3.5 cm from the anus. We pretreated BDNF^+/+^ mice with recombinant TrkB Fc chimera, TrkB/Fc (10 ng/mouse, intracolonical infusion, 30 min before FSN infusion, R&D, USA)^12^ and fluorocitrate (20 μmol/kg, i.p, twice a day at 9 am and 6 pm for 7 days, sigma)^51^ to block the effect of colonic BDNF and EGCs in BDNF^+/+^ mice, respectively.

CRD started at 1 hour after the intracolonic infusion of FSN under pressures of 15, 30, 45 and 60 mmHg (Supplementary Methods-6). The EMG activity was used to evaluate the visceromotor response of mice and was recorded by the Powerlab BL-420E system. Data as expressed by the area under the curve were analyzed by Labchart v5.0 software. EMG activity was corrected for the baseline activity and expressed as “% of baseline”.

#### Immunostaining and western blotting for BDNF, GFAP, TrkB and SP in mice

Colonic samples (at the infused area) were obtained from mice 4 hours after supernatants infusion. We used western blotting or immunohistochemistry to detect the expressions of mucosal BDNF, GFAP, TrkB and SP levels.

### EGC cell line and mesenteric afferent nerve recording

Rat EGC cell line, CRL-2690, was purchased from American Type Culture Collection, (ATCC, Manassas, VA, USA). Cells were cultured in high-glucose Dulbecco’s modified Eagle’s medium (DMEM, Gibco by Invitrogen, CA, USA), supplemented with 10% FBS (Gibco) under conditions of 5% CO_2_ at 37 °C. CRL-2690 cells were seeded in 6-well plates and exposed to recombinant human BDNF (r-HuBDNF, 0–200 ng/ml, Peprotech, Rocky Hill, USA) for 2 h. After obtaining the optimal incubating dose (100 ng/ml), we used phospholipase Cγ1 (PLC-γ1) inhibitor (U73122, 2 μM for 30 minutes; Sigma-Aldrich, St. Louis, MO) or TrkB/Fc, (2.5 μg/ml for 30 minutes) to previously block the r-HuBDNF-TrkB pathway.

#### Western blotting

SP and signaling pathway proteins in r-HuBDNF -stimulated EGCs were detected by western blotting. Cells were treated with r-HuBDNF (100 ng/ml) for 2 h in the presence of Brefeldin A (used for inhibiting protein release; 3 μg/ml, eBioscience, San Diego, CA, USA). Following our standard protocol for western blotting, GFAP, TrkB, total PLC-γ1, phosphorylated-PLCγ1 (p-PLCγ1), and SP were detected and quantified (Supplementary Methods-7).

#### Mesenteric afferent nerve recording

Mesenteric afferent nerve recording was performed to investigate the effects of supernatants collected from r-HuBDNF-stimulated CRL-2690 cells or r-HuBDNF alone on nerve excitability and mechanosensitivity (Supplementary Methods-8). r-HuBDNF (10–200 ng/ml), SP (10^−12^–10^−4 ^M, Sigma Chemical Co, St Louis, USA) or supernatants or DMEM were administered in the organ bath after the baseline recording was stable for 20 min or after preincubation with TrkB/Fc (2.5 μg/ml) or FK888 (4 × 10^−6^ M), or TrkB/Fc (2.5 μg/ml) plus FK888 (4 × 10^−6^ M) for 15 min. To detect mechanosensitivity, continuous ramp distention was performed to let the jejunal segment distend up to 60 cmH_2_O for approximately 90 s. Then the intraluminal pressure returned to the baseline. Spike discharge frequency (impulse/s) was recorded and analyzed by Spike-2 software to evaluate the afferent nerve response to stimulation. There were 15 minutes between two adjacent stimuli for the nerve recovering to baseline discharge.

### Statistics

Using GraphPad Prism 6.0c software, data were presented as the mean values ± the standard deviation (SD), expect for fecal protease activity, which was expressed as the median (interquartile range; IQR). Two-group differences were determined by an independent Student’s t test and multiple comparisons by a one-way ANOVA test (including Bonferroni’s post-hoc multiple comparisons). The relationship between protein levels and the severity/frequency of abdominal pain in patients with IBS was assessed by Spearman rank correlations. Two-tailed *P* values < 0.05 were taken as a significant difference.

## Additional Information

**How to cite this article**: Wang, P. *et al*. BDNF contributes to IBS-like colonic hypersensitivity via activating the enteroglia-nerve unit. *Sci. Rep*. **6**, 20320; doi: 10.1038/srep20320 (2016).

## Supplementary Material

Supplementary Information

## Figures and Tables

**Table 1:** Characteristics of study subjects.

	Healthy controls	IBS	*P* value	IBS-D	IBS-C	*P* value
	30	30	—	20	10	—
Sex ratio (M/F)	14/16	12/18	0.60	9/11	3/7	0.43
Mean age (years)	44.8 ± 10.4	49.3 ± 13.4	0.22	51.1 ± 11.9	44.4 ± 14.7	0.27
Abdominal pain scores						
Severity	0.10 ± 0.31	2.30 ± 0.59	0.000	2.35 ± 0.59	2.20 ± 0.63	0.53
Frequency	0.10 ± 0.30	2.27 ± 0.69	0.000	2.30 ± 0.73	2.20 ± 0.63	0.72
HAD depression scale	2.50 ± 1.74	2.67 ± 1.06	0.66	2.65 ± 0.88	2.70 ± 1.42	0.90
HAD anxiety scale	2.70 ± 1.47	2.47 ± 1.28	0.51	2.50 ± 1.50	3.00 ± 1.49	0.40

IBS: irritable bowel syndrome.

Data are expressed as the mean ± SD.

**Table 2:** Quantitative assessment of ultrastructural alterations of EGCs.

Per cell	Healthy controls	IBS	*P* value
Heterochromatin (IOD/Area)	0.36 ± 0.07	0.29 ± 0.07	0.009
Mitochondrion (n)	4.20 ± 0.83	8.05 ± 1.35	0.000
Glycogenosome (n)	23.58 ± 6.13	30.05 ± 9.32	0.016

EGC: enteroglial cell.

IOD: integrated optical density.

n: number: Data are expressed as the mean ± SD.
