# Author Correction: BDNF contributes to IBS-like colonic hypersensitivity via activating the enteroglia-nerve unit

**DOI:** 10.1038/s41598-021-98826-8

**Published:** 2021-09-21

**Authors:** Peng Wang, Chao Du, Fei-Xue Chen, Chang-Qing Li, Yan-Bo Yu, Ting Han, Suhail Akhtar, Xiu-Li Zuo, Xiao-Di Tan, Yan-Qing Li

**Affiliations:** 1 Department of Gastroenterology, Qilu Hospital, Shandong University, Jinan, 250012 People’s Republic of China; 2 Laboratory of Translational Gastroenterology, Qilu Hospital, Shandong University, Jinan, 250012 People’s Republic of China; 3 Department of Physiology, Shandong University School of Medicine, Jinan, 250012 People’s Republic of China; 4 Department of Pediatrics, Feinberg School of Medicine, Northwestern University, Chicago, IL 60611 USA

Correction to: *Scientific Reports* 10.1038/srep20320 published online 03 February 2016

This Article contains errors.

In Figure 3H, the figures for the control group (BDNF+/+ mice) were mistakenly selected from the candidate representative images for IBS-D FSN group (BDNF+/+ mice).

The correct Figure 3 and its accompanying legend appear below.

**Figure 3:**
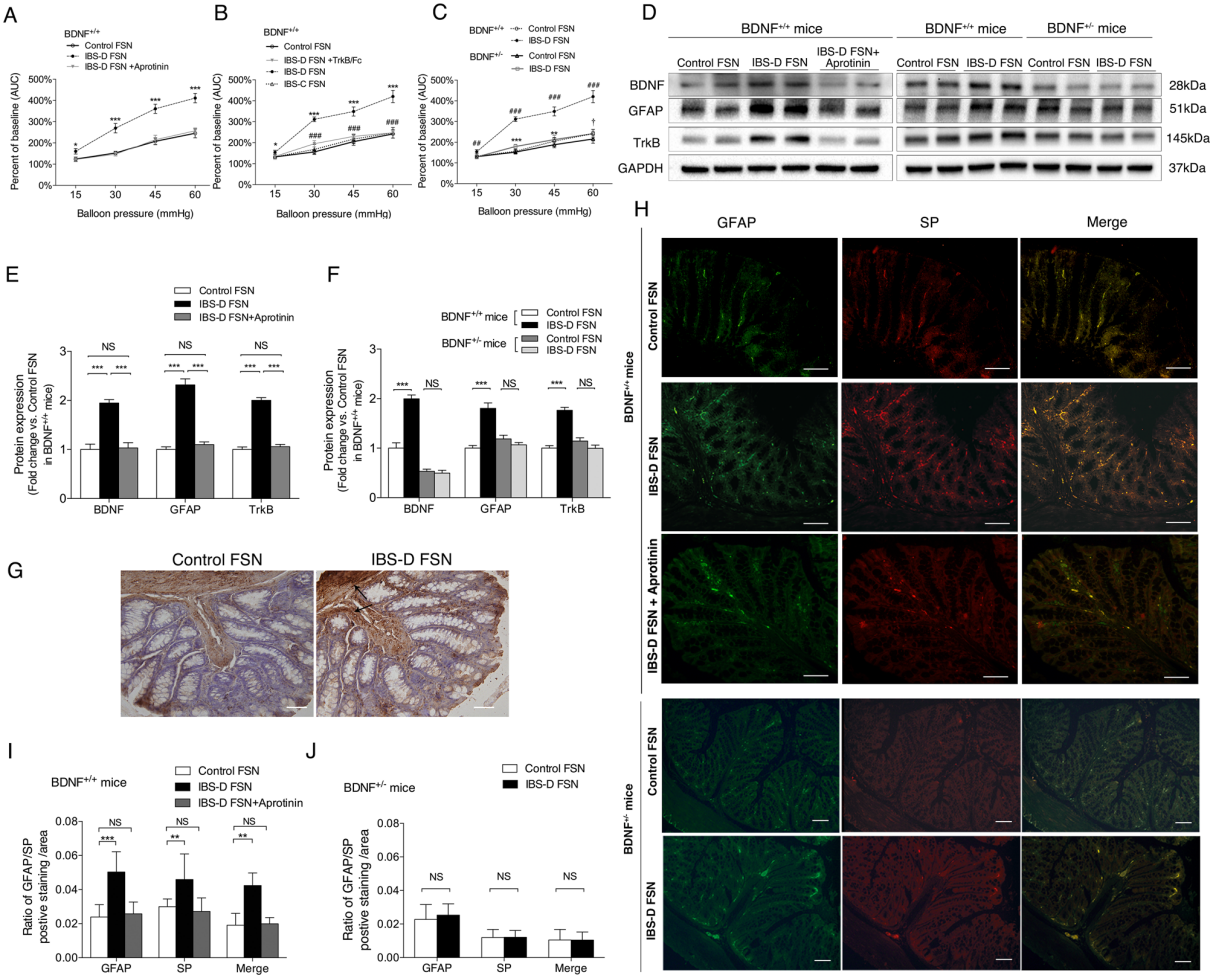
Alterations of visceral sensitivity and expressions of BDNF, GFAP and substance P in colon of BDNF^+/+^ and BDNF^+/−^ mice in the presence or absence of IBS fecal supernatants (FSN) stimulation. (**A**) Intracolonic infusion of IBS-D FSN significantly increased electromyographic (EMG) activity of abdominal muscles in wild-type mice (*, IBS-D FSN vs. control). (**B**) TrkB/Fc significantly suppressed the effect of IBS-D FSN at 30, 45 and 60 mmHg (*, IBS-D FSN vs. control; #, IBS-D FSN vs. IBS-D FSN plus TrkB/Fc). (**C**) The overall response to the CRD after treatment by IBS-D FSN in BDNF^+/−^ mice was significantly lower than that in BDNF^+/+^ mice. (*, IBS-D FSN vs. control FSN in BDNF^+/−^ mice; #, IBS-D FSN in BDNF^+/+^ mice vs. in BDNF^+/−^ mice; ^†^control FSN in BDNF^+/+^ mice vs. in BDNF^+/−^ mice). (**D**–**F**) Western blotting analysis of BDNF, GFAP and TrkB protein levels in colon of BDNF^+/+^ and BDNF^+/−^ mice 4 h after intracolonic infusion with IBS-D FSN compared with control FSN or aprotinin-pretreated IBS-D FSN. (**G**) Overview of GFAP expression in the full-thickness colonic wall in BDNF^+/+^ mice 4 h after intracolonic infusion with IBS-D FSN compared with control FSN. Black arrow shows increased expression of GFAP in muscular layers. (**H**) Dual immunofluorescence staining of SP and GFAP in colon of BDNF^+/+^ and BDNF^+/−^ mice 4 h after intracolonic infusion with IBS-D FSN compared with control FSN or aprotinin-pretreated IBS-D FSN. Co-immunostaining for colonic SP and GFAP was significantly increased in BDNF^+/+^ mice (**I**) but not in BDNF^+/−^ mice (**J**) after 4-hour intracolonic incubation with IBS-D FSN. The results are displayed as the mean ± SD. (^*^P < 0.05 ^**^P < 0.01, ^***^P < 0.001, ^##^*P* < 0.01, ^###^*P* < 0.001, ^†^*P* < 0.05; AUC, area under curve; NS, not significant; target protein levels were normalized to GAPDH; n = 8 per group; scale bars: 50 μm). The gels were run under the same experimental conditions. Cropped gels/blots are presented. (Full-length gels/blots are shown in Suppl. Fig. S2 with indicated cropping lines.)

These changes do not affect the conclusions of the article.

