# Supplementary material for: BDNF contributes to IBS-like colonic hypersensitivity via activating the enteroglia-nerve unit
Source: Sci Rep. 2016 Feb 3;6:20320. doi: 10.1038/srep20320 (PMC4738267; doi:10.1038/srep20320)
Supplement: Supplementary Information [file srep20320-s1.pdf]

BDNF contributes to IBS-like colonic hypersensitivity via activating the enteroglia-nerve unit

Peng Wang[1, 2], Chao Du[1, 2], Fei-Xue Chen[1, 2], Chang-Qing Li[1, 2], Yan-Bo Yu[1, 2], Ting Han[3], Suhail Akhtar [4], Xiu-Li Zuo[1, 2], Xiao-Di Tan[4], Yan-Qing Li[1, 2]

[1] Department of Gastroenterology, Qilu Hospital, Shandong University, Jinan 250012, P. R. China

[2] Laboratory of Translational Gastroenterology, Qilu Hospital, Shandong University, Jinan 250012, P. R. China

[3] Department of Physiology, Shandong University School of Medicine, Jinan 250012, P. R. China

[4] Department of Pediatrics, Feinberg School of Medicine, Northwestern University, Chicago, Illinois 60611, USA

Corresponding author

Yan-Qing Li, M.D., PH.D.

Department of Gastroenterology, Qilu Hospital, Shandong University, Jinan 250012, China

E-mail: liyanqing@sdu.edu.cn

Tel: +86-531-82169236

Fax: +86-531-82169236

Supplementary Figures

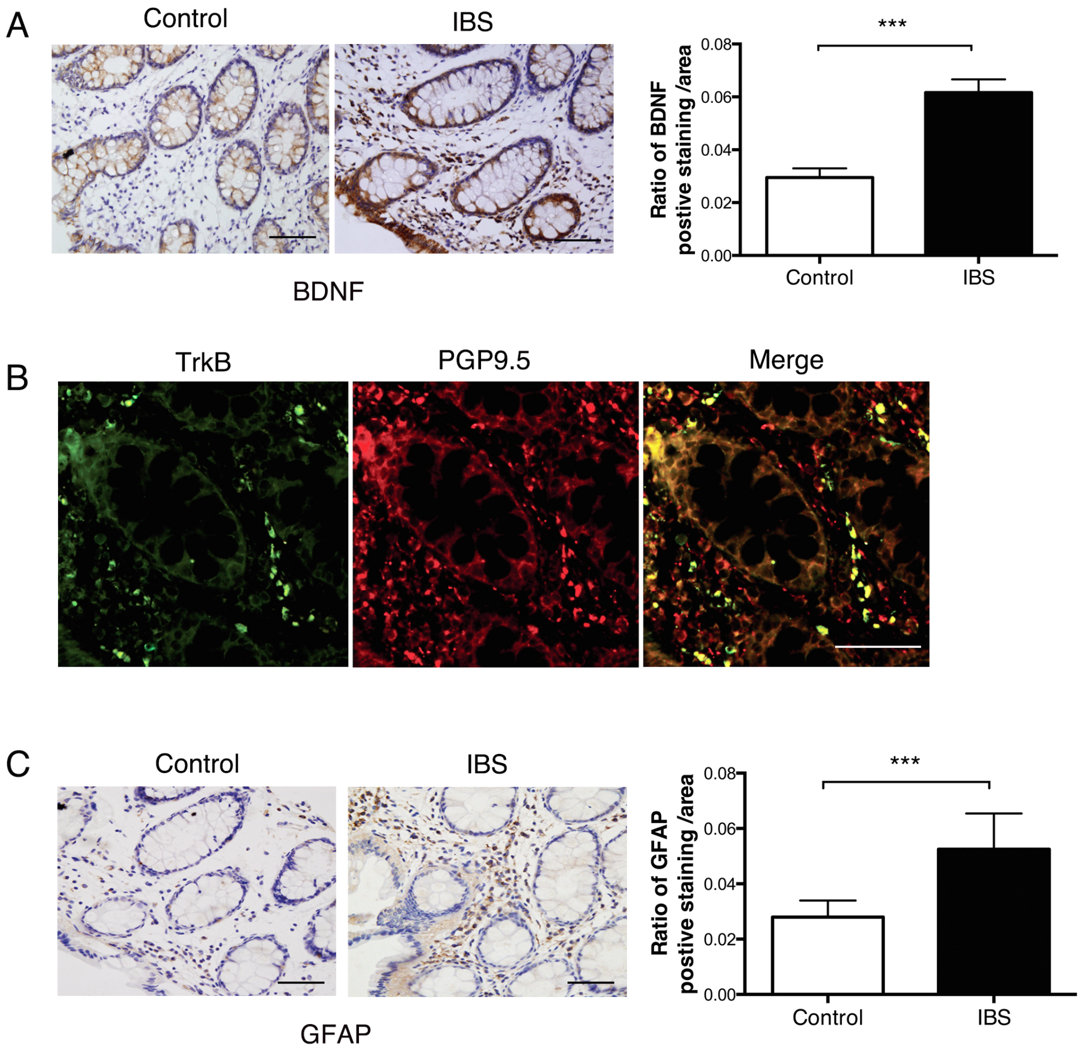

Supplementary Figure S1

A. Immunohistochemistry staining shows overall expression of BDNF in the colonic biopsy specimens of IBS patients and HCs. n=30 for IBS, n=30 for HCs; scale bars: 50 µm

B. Co-localization of TrkB and nerve fibers in colonic mucosa. Scale bars: 50 µm

C. Immunohistochemistry staining represents overall expression of GFAP in the

colonic biopsy specimens of IBS patients and HCs. n=30 for IBS, n=30 for HC; scale bars: 50 μm

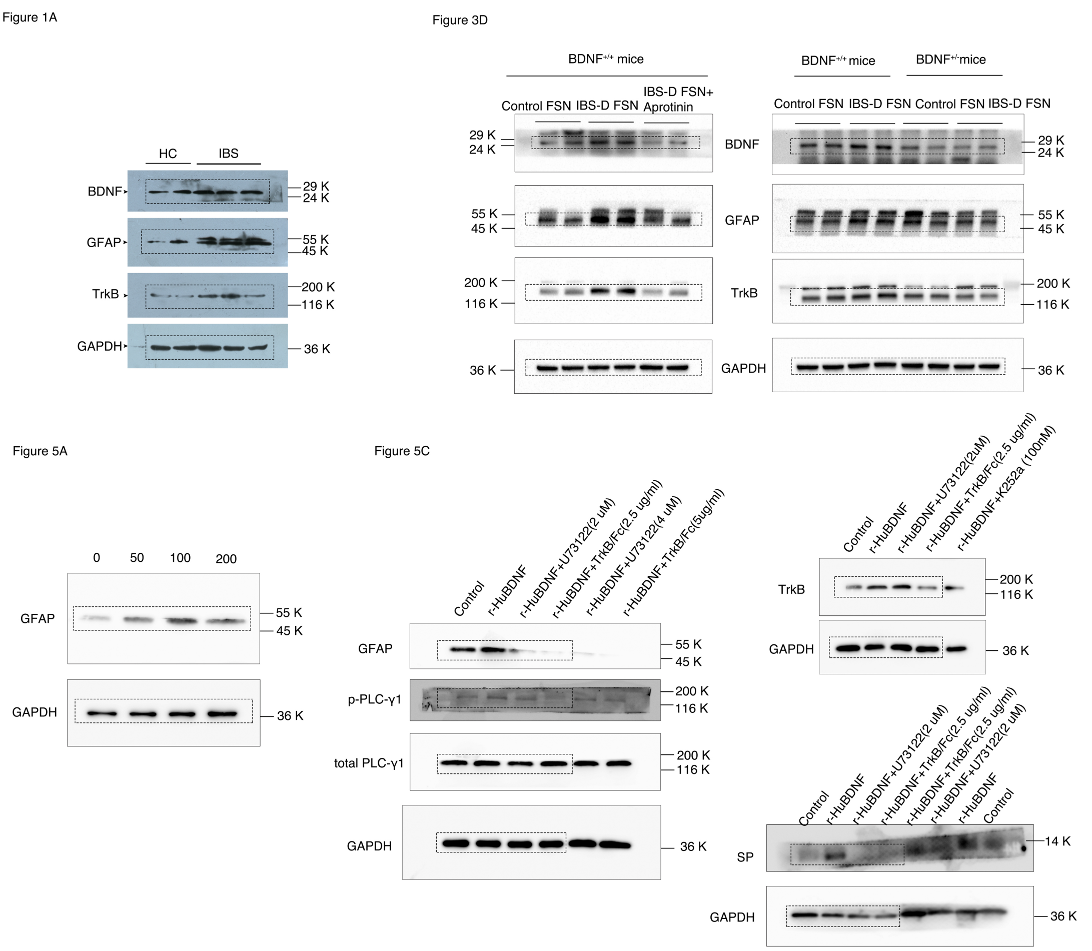

Supplementary Figure S2

Full scan images of the immunoblots. In several cases, selected data were shown in the manuscript and the PVDF membranes for immunoblots were cut into strips to minimize the amount of antibodies that are necessary for analysis. Scans of entire PVDF strips are provided.

Supplementary methods

Supplementary methods-1

BDQ scoring system

In this questionnaire, the severity and frequency of abdominal pain/discomfort over the last 2 weeks were the key parameters in this study. Symptom score was graded 0 - 4 according to the influence of symptom on patients' daily activities: 0, absent; 1, mild (not influencing activities); 2, relevant (diverting from but not urging modification of activities); 3, severe (influencing activities markedly enough to urge modifications); 4, extremely severe (precluding daily activities). Similarly, symptom frequency was also graded 0 - 4 (0, absent; 1, up to 1 day/week; 2, 2 or 3 days/week; 3, 4-6 days/week; 4, daily).

Supplementary methods-2

Briefly, we incubated IBS-D FSN (25 μl) with 1 ml of reaction buffer (20 mM Tris-HCl and 0.15 M NaCl; PH 8.3) and 1 ml of 0.5% (w/v) azo-casein at 40℃. Trichloracetic acid (1 ml; 10% (v/v)) was used to stop the reaction after 20 min, followed by centrifugation. Supernatants were collected from the reaction mixture for

absorbance measurement at 366 nm.

Supplementary methods-3

Immunohistochemistry, immunofluorescence and western blotting

Biopsies for histology, immunohistochemistry were immediately fixed in 10% buffered formalin. Those for western blotting were snap frozen in liquid nitrogen and stored at -80℃.

For immunohistochemistry, paraffin-embedded tissues were cut into 4-μm-thick sections, followed by antigen unmasking process, and incubated overnight at 4 ℃ with BDNF rabbit antibody (1/200 dilution; Abcam, Cambridge, UK) or GFAP mouse antibody (1/100 dilution; Millipore, Billerica, MA, USA). Phosphate buffered saline replaced the primary antibody as negative control. The subsequent detection was use of anti-rabbit or mouse immunohistochemistry assay kit (DAKO, Carpentaria, CA, USA) as a chromogen for visualization. Hematoxylin was used to counterstain the nuclei. All the slides were viewed and photographed under microscope (Olympus Bx51) combined with a digital camera (Olympus).

For immunofluorescence, 4-μm-thick sections were incubated overnight with a

mixture of GFAP mouse antibody (1/100; Millipore) and TrkB rabbit antibody (1/200 dilution; Santa, Cruz, CA, USA)/substance P rabbit antibody (1:100; PL Laboratories, British Columbia, Canada)/PGP9.5 rabbit antibody (1/100, Bioworld, Atlanta, Georgia, USA), or a mixture of TrkB rabbit antibody (1/200 dilution; Santa, Cruz, CA, USA) and PGP9.5 mouse antibody (1:200 dilution, Abcam, Cambridge, UK). Rhodamine-conjugated goat anti-rabbit/mouse IgG antibody (1:100; Zhongshan Gold Bridge, Beijing, China) and a FITC-conjugated goat anti-mouse/rabbit antibody (1:100; Zhongshan Gold Bridge) were used to detect the above primary antibodies respectively. Additional controls (Phosphate buffered salines in place of the primary antibodies) were conducted to exclude cross-reaction during double labeling. Slides were detected using a fluorescence microscope (Olympus IX-70). We chose five 40x magnification fields per tissue section at random, and two independent blinded observers obtained the mean area values of positive signals for final analysis by using the Image-Pro Plus 6.0 software.

For western blotting, total proteins were isolated from the colonic tissues in radioimmuno-precipitation assay (RIPA) buffer (Beyotime Institute of Biotechnology,

Nanjing, Jiangsu, China), and quantified with a BCA protein quantification kit (Beyotime). Protein (20 μg) from each sample was separated by SDS-PAGE and transferred to a PVDF membrane (0.22 μm pore; Millipore). Membranes were blocked with 5% skim milk for 1h, and then incubated with primary antibodies (BDNF rabbit antibody, 1/500 dilution, Abcam; GFAP mouse antibody, 1/500 dilution, Millipore; TrkB rabbit antibody, 1/1000, Cell signaling Technology, USA) at 4 ℃ overnight, and followed by incubation with horseradish peroxidase-conjugated anti-rabbit or mouse secondary antibodies (1:10000, Zhongshan Gold Bridge) for 1 hour. Protein bands were detected by an enhanced chemiluminescent substrate (Millipore) on Kodak biomax light film or by chemiluminescence reader ImageQuant LAS4000 (GE, USA). Density of the protein bands was analyzed by Image J software (v1.47c. Bethesda, MD, USA). Targeted proteins were normalized to GAPDH (mouse antibody, 1:1000, Beyotime).

Supplementary methods-4

Procedure for TEM samples preparation

Briefly, biopsy specimens were prefixed in cacodylate-buffered 2.5%

glutaraldehyde solution, postfixed with osmium tetroxide, and then dehydrated, infiltrated and embedded in araldite. Semi-thin (590 nm thick) sections were stained with 0.25% Toluidine-blue and screened with an optical microscope to observe EGC in mucosa. Following this, ultra-thin (90 nm thick) sections were stained with 4 % uranylacetate, and then with 0.4% lead citrate. Photographs were magnified from ×15000 to ×30000 (at least 5 fields at ×15,000, 5 fields at ×30000) under a JEOL CX1200 electron microscope.

Supplementary Methods-5

Briefly, we took two silver electrodes, coated by sterile plastic tube (inside diameter: 0.5 mm) for implantation. One end of each electrode was implanted into the abdominal external oblique muscle (0.5 cm between the two electrodes) under 10% chloral hydrate (350 mg/kg, intraperitoneally) anesthesia, and the other two ends were exteriorised from the back of the mice neck via a subcutaneous tunnel for connection to an amplifier.

Supplementary Methods-6

Mice were kept in a small plastic chamber (4.5 cm in diameter and 10 cm in

length) to restrict their movement. A polyethylene catheter (1 cm polyethylene balloon secured to one end) was inserted into the descending colon, 1 cm proximal to the anus. The catheter was taped to the tail to hold the balloon in position. After mice were fully awake and recovered from isoflurane anesthesia, the balloon was progressively inflated in 15 mmHg steps, from 0 to 60 mmHg (15, 30, 45 and 60 mmHg), each step lasting 10 s with a 5 min non-distension interval and repeated 3 times.

Supplementary Methods-7

Detailed primary antibodies are as follows: GFAP (mouse-derived antibody, 1/500, Millipore, Billerica, MA, USA), TrkB (rabbit-derived antibody, 1/1000, Cell signaling Technology, USA), PLC-γ1 (rabbit-derived antibody, 1/1000, Cell signaling Technology, USA), phosphorylated-PLC-γ1 (rabbit-derived antibody, 1/1000, Cell signaling Technology, USA) and SP (rabbit-derived antibody, 1/500, PL Labratories, British Columbia, Canada) were detected respectively.

Supplementary Methods-8

For mesenteric afferent nerve recording procedure, a segment of proximal

jejunum (about 3 cm in length) with attached mesentery was isolated carefully from isoflurane-anesthetized rat. The whole segment was placed in a custom-made organ bath that consisted of a perfusion chamber (intestinal segment fixed in here) and a recording chamber (dissected mesenteric nerve connected to a pair of bipolar platinum recording electrodes here). Kreb's buffer (composition (mmol/L): K^+5.9, Na^+ 143.5, Cl^- 126, Mg^{2+} 1.2, Ca^{2+} 2.5, SO4 1.2, HCO_3^- 25 H_2PO_4 1.2; glucose 10 and sodium buyrate 1, pH 7.0, temperature 32℃) oxygenated with O_2/CO_2 was continuously perfused into the intestinal segment (perfusion rate: 5ml/min) and suspended when stimulation was administered. The multi-unit afferent signal was captured and recorded by a Cambridge Electronic Design single channel 1902 preamplifier/filter (Cambridge, UK), amplified and filtered, and then transferred to a power Micro 1401 interface system.
